# Observation of anomalous Hall effect in a non-magnetic two-dimensional electron system

**DOI:** 10.1038/ncomms14777

**Published:** 2017-03-16

**Authors:** D. Maryenko, A. S. Mishchenko, M. S. Bahramy, A. Ernst, J. Falson, Y. Kozuka, A. Tsukazaki, N. Nagaosa, M. Kawasaki

**Affiliations:** 1 RIKEN Center for Emergent Matter Science (CEMS), Wako 351-0198, Japan; 2 Department of Applied Physics and Quantum Phase Electronics Center (QPEC), University of Tokyo, Tokyo 113-8656, Japan; 3 Max-Planck-Institut fr Mikrostrukturphysik, Weinberg 2, 06120 Halle, Germany; 4 Institut für Theoretische Physik, Johannes Kepler Universität, A 4040 Linz, Austria; 5 Institute for Materials Research, Tohoku University, Sendai 908-8577, Japan

## Abstract

Anomalous Hall effect, a manifestation of Hall effect occurring in systems without time-reversal symmetry, has been mostly observed in ferromagnetically ordered materials. However, its realization in high-mobility two-dimensional electron system remains elusive, as the incorporation of magnetic moments deteriorates the device performance compared to non-doped structure. Here we observe systematic emergence of anomalous Hall effect in various MgZnO/ZnO heterostructures that exhibit quantum Hall effect. At low temperatures, our nominally non-magnetic heterostructures display an anomalous Hall effect response similar to that of a clean ferromagnetic metal, while keeping a large anomalous Hall effect angle *θ*_AHE_≈20°. Such a behaviour is consistent with Giovannini–Kondo model in which the anomalous Hall effect arises from the skew scattering of electrons by localized paramagnetic centres. Our study unveils a new aspect of many-body interactions in two-dimensional electron systems and shows how the anomalous Hall effect can emerge in a non-magnetic system.

## Abstract (web summary)

The realization of the anomalous Hall effect in high-mobility two dimensional electron systems has so far remained elusive. Here, the authors observe its emergence in MgZnO/ZnO heterostructures and attribute it to skew scattering of electrons by localized paramagnetic centres.

A magnetic field *B* applied perpendicularly to the charge carrier flow deflects the carriers upon the acting Lorentz force. In solids, this results in the carrier accumulation at the boundaries of the system until the built-up electric field *E*_Hall_ compensates for the transverse flow of charge carriers (Fig. 1a, left). This mechanism, so-called ordinary Hall effect, gives rise to the Hall voltage *U*_Hall_ being linearly proportional to *B*. In systems with both spin–orbit coupling and spontaneous ferromagnetic polarization, the mobile charge carriers gain an additional transverse momentum (Fig. 1b). Consequently, the built-up Hall voltage attains a component *U*_AHE_, so-called anomalous Hall component, originating from the spin–orbit interaction and being proportional to the magnetization *M*_*z*_ (refs 1, 2, 3, 4, 5; Fig. 1a, right). The total Hall effect, conventionally given by the Hall resistance *R*_*yx*_, is then composed as:


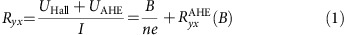


where *I* is the current flowing through the system, *e* is the elementary charge, *n* is the charge carrier density, and 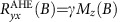, where *γ* establishes the relation between the anomalous Hall resistance 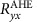 and *M*_*z*_. Anomalous Hall effect (AHE) is a well-established phenomenon in intrinsically ferromagnetic metals as well as in the induced ferromagnetic degenerated semiconductors, known as diluted magnetic semiconductors (Fig. 1b). Common to both material classes is the scaling behaviour of the anomalous Hall conductivity 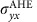 with the sample conductivity 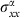, where *α* is the scaling power factor^6^,^7^. Most materials show the power factor of either *α*≈1.6 or 0.0, whereas *α*≈1.0 is only rarely observed in the very clean ferromagnetic metals^8^. Such a scaling behaviour has been theoretically proposed to be reproducible in ferromagnetic systems with dilute impurities^9^. According to these models, *α*=1.6 is a characteristics of a system with a large number of scattering centres. As this number reduces *α* changes to 0, and in the clean limit, extrinsic scattering regime, *α*=1.0, is expected. Since this common scenario takes place in ferromagnets, the realization of AHE in a nominally non-magnetic system is counter-intuitive.

Here we report the observation of such an unusual effect in a high-mobility non-magnetic two-dimensional electron system formed at the interface MgZnO and ZnO, which shows both integer and fractional quantum Hall effects^10^,^11^,^12^. The observed AHE exhibits the scaling power factor *α*≅1 at low temperatures and keeps a large AHE angle *θ*_AHE_≈20° (refs 7, 8, 9; Fig. 1d). Our observation is consistent with the Giovannini–Kondo (GK) model for AHE, whose mechanism sketched in Fig. 1c considers the coupling of localized magnetic moments **J** with the orbital momentum **l** of mobile electrons leading to the skew scattering amplitude **J**·(**k** × **k**′) (refs 13, 14, 15, 16). In particular, the model can successfully explain *α*≅1, linear *B* dependence of 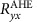 at low field and 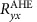 saturation at high field. Moreover, it does not contradict to the observation of positive magnetoresistance, which is not expected in conventional cases of AHE in ferromagnets. We are not aware of another theory that can simultaneously cover all mentioned aspects of our experimental results.

## Results

### Transport characteristics of MgZnO/ZnO heterostructure

**Figure 2:**
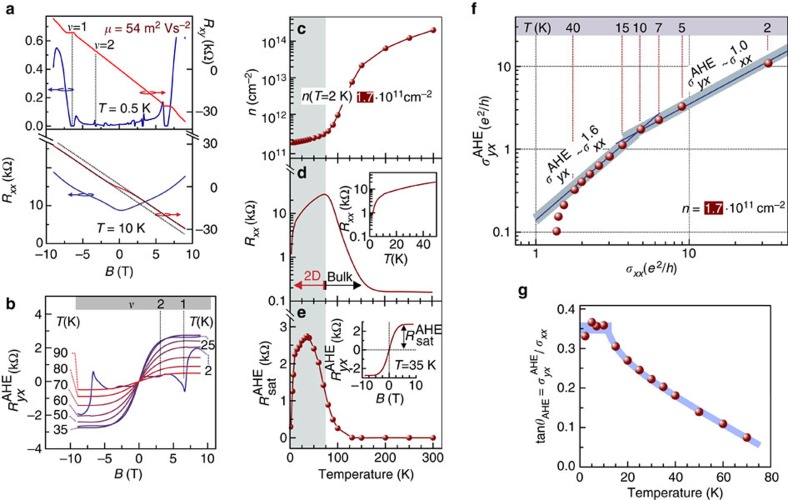
Sample characteristics establish the anomalous Hall effect. (**a**) Magnetotransport *R*_*xx*_ and *R*_*yx*_ at two representative temperatures. (**b**) Anomalous Hall effect component at different temperatures. The Landau level integer filling factors *ν* becomes visible at *T*<8 K. (**c**) Temperature dependence of the charge carrier density extracted from the ordinary Hall effect in high field. (**d**) Four-point resistance *R*_*xx*_ as a function of temperature. (**e**) Temperature dependence of the saturated value of anomalous Hall effect resistance 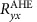. (**f**) AHE scaling: 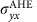
∝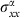. *α*=0.94±0.08 is observed between 2 and 10 K, whereas *α*>1 at higher temperature. (**g**) AHE angle increases with the decreasing temperature.

MgZnO/ZnO heterostructures studied in this work are grown with the oxide molecular beam epitaxy technique without incorporating any magnetic elements and cover a charge carrier density range between 1.7 × 10^11^ and 18 × 10^11^ cm^−2^ (ref. 17). Secondary ion mass spectroscopy of a typical high-mobility heterostructure confirms the absence of the undesired impurities including magnetic (Supplementary Note 1). Two-dimensional charge carriers are paramagnetic and one observes an alternating sequence of Landau levels with up and down spin orientations, as it was confirmed by the observation of multiple Landau level coincidence events^12^,^18^. At low temperatures, the samples studied in this work exhibit a conventional magnetotransport for MgZnO/ZnO heterostructures, with integer and fractional quantum Hall states as exemplified in Fig. 2a for a sample with *n*=1.7 × 10^11^ cm^−2^ (refs 10, 11, 12). We note that the incorporation of magnetic moments in other high-mobilty two-dimensional charge carrier systems leads frequently to the deterioration of the device performance compared to non-doped structures^19^,^20^,^21^,^22^,^23^. The sample shown in Fig. 2a is now discussed in detail, whereas the characteristics' comparison of all samples follows later. At elevated temperature and high magnetic field *B*, the Hall resistance is linearly proportional to *B* and therefore one evaluates the charge carrier density *n*=Δ*R*_*yx*_/*e*Δ*B*. While *n* decreases by three orders of magnitude between 300 and 75 K as shown in Fig. 2c, the longitudinal sample resistance *R*_*xx*_ displayed in Fig. 2d increases gradually in the same temperature range. This behaviour is the result of bulk charge carrier freeze-out in ZnO substrate^24^. Below 75 K, the interface, that is, 2D, conductance prevails, whereby it is characterized by a decreasing *R*_*xx*_ concomitant with a marginal change in *n*. In the low magnetic field region, *R*_*yx*_ shows the non-linear behaviour at *T*⩽90 K, as the lower panel of Fig. 2a illustrates it for *T*=10 K. Such non-linearity cannot be explained with a two-band conductance model with electron carriers in each channel (Supplementary Note 2). Therefore, the crossover from three-dimensional to 2D transport, as shown in Fig. 2c,d, cannot lead to such non-linearity. Neither the electron interaction effects can fully account for the non-linearity^25^,^26^,^27^ (Supplementary Note 3). Nor the electron bulk localization, which are important for the appearance of quantum Hall effect, are in play in our system^28^. Furthermore, the quantum Hall effect takes place only at particular values of the magnetic field and does not cover the full magnetic field range used in our experiment. Rather the non-linear *R*_*yx*_ is the manifestation of the AHE in ZnO. The AHE component is extracted by subtracting the ordinary Hall effect component from the raw data according to equation (1) and displayed in Fig. 2b for several temperatures. Above some field *B*^sat^, which depends on the temperature, 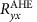 remains field-independent, thus reaching the regime of saturation. This saturated AHE resistance 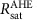 has a non-trivial temperature dependence shown in Fig. 2e and appears in the temperature regime with a dominating 2D transport, suggesting that the AHE is associated with the 2D transport rather than with the bulk transport. The temperature dependence of 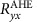 follows the temperature dependence of sample resistance *R*_*xx*_—both decrease with the lowering temperature (*T*<50 K), which is therefore consistent with the AHE scaling behaviour^1^,^6^,^7^,^9^.

### AHE scaling

To analyse the scalability of the anomalous Hall conductance 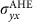 with the sample conductance *σ*_*xx*_, the two parameters are conventionally calculated for each temperature^6^:


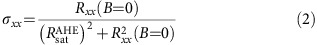



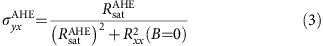


Figure 2f displays 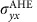 versus *σ*_*xx*_ on a double logarithmic scale. Striking is the observation of scaling 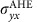
∝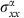 with *α*=0.94±0.08 for *T*⩽10 K (Supplementary Note 4). Conventionally, *α*=1 is associated with the skew-type electron scattering, which is understood as the asymmetric scattering for electrons with up and down spin orientations^29^,^30^. At higher temperature, *α* increases gradually and in a limited temperature range *α*=1.6 is apparent. The AHE angle tan(*θ*_AHE_)=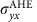/*σ*_*xx*_, shown in Fig. 2g increases with the decreasing temperature and reaches a constant value tan(*θ*_AHE_)=0.35 for *T*<10 K. This temperature dependence of *θ*_AHE_ substantiates that the AHE does not vanish at low temperatures, although one might falsely be inclined to interpret a vanishing AHE at low temperature based on temperature dependence of 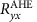 displayed in Fig. 2e. As the temperature decreases below *T*=2 K *σ*_*xx*_ keeps increasing due to the increase of the electron mobility^17^. Consequently, 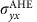 component increases because of the scaling 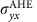
∝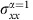, so that its contribution to the Hall resistance *R*_*yx*_ at low temperature becomes more difficult to resolve. This explains why the Hall effect at ^3^He temperature and at the temperature of dilution refrigerator, at which the most of our previous studies have been conducted before, appears linear in the low magnetic field^10^,^11^,^12^. More importantly, non-vanishing AHE angle *θ*_AHE_ implies that the scattering mechanism leading to the AHE can sustain down to low temperature.

### Magnetic properties

The AHE can arise in our system when the mobile electrons interact with the magnetic moments being polarized by the external magnetic field. In the absence of the external magnetic field, the AHE current is zero. However, the polarization of the localized magnetic moments by the application of the magnetic field induces the polarization of angular orbital momentum of conduction electron leading to a nonzero AHE^15^. The magnetic moments are likely to be the point defects in the epitaxial ZnO with localized unpaired electrons^31^,^32^,^33^,^34^ (Supplementary Note 5). Although the molecular beam epitaxy enabled to reduce the number of defects with thereof resulting high electron mobility, the defects cannot be completely avoided.

**Figure 3:**
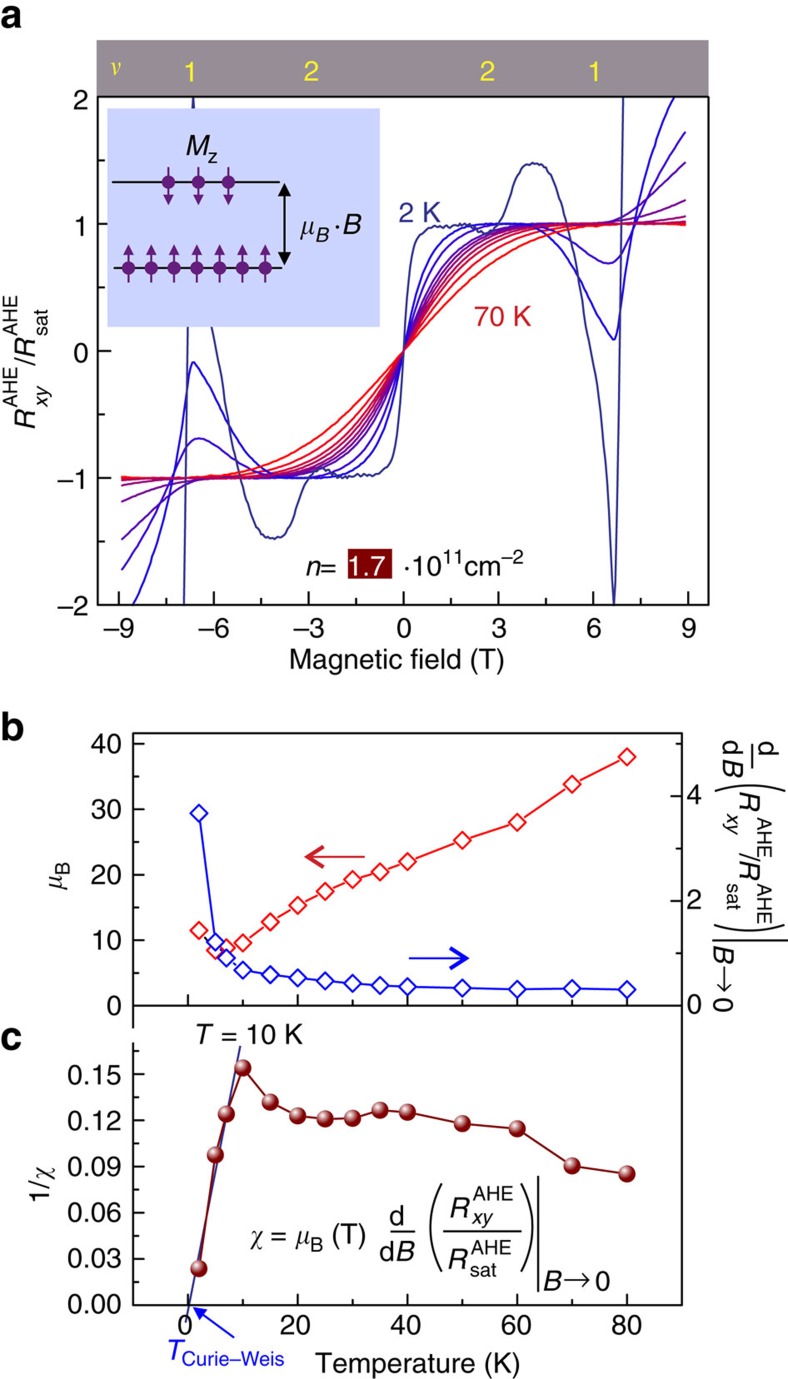
Magnetic characteristics deduced from electrical transport. (**a**) Temperature dependence of the anomalous Hall effect normalized to the saturated value of AHE. Such a representation makes apparent the similarity with the magnetization behaviour of a paramagnetic system. The Brillouin function describes well the field dependence of AHE. (**b**) Left axis: temperature dependence of *μ*_B_ obtained from the fitting the AHE traces in **a** with the Brillouin function. Right axis: temperature dependence of the slope of AHE around *B*=0 in **a**. (**c**) Magnetic susceptibility *χ* obtained from the slope of AHE and temperature dependence *μ*_B_. For *T*<10 K, the dependence of 1/*χ* on *T* can be approximated with the Curie–Weiss law (black line).

We note that, because of 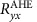
=*γM*_z_(*B*), the character of the magnetic moments can be deduced from the analysis of the AHE field dependence displayed in Fig. 2b. It turns out that the Brillouin function, which describes the magnetization of a paramagnetic system in an external magnetic field, depicts well the field dependence of AHE for all temperatures of the experiment when assuming *g*=2 and *J*=1/2 (Supplementary Note 6). Such a description reveals that the system of localized magnetic moments is characterized by an extremely large effective magnetic moment *μ*_B_ shown on the left axis in Fig. 3b and is therefore qualified as a superparamagnet. The large *μ*_B_ reflecting the value of magnetic moment averaged over the sample, rather than the on-site value of *μ*_B_, is on itself not very surprising, since both the unconventionally large values and temperature dependence of magnetic moments have also been obtained, for instance, in the studies of LaCoO_3_ and was explained with the formation of polarons^35^. Furthermore, the fact that 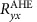 is described with the Brillouin function, points to the polarization of localized magnetic moments, rather than the Pauli magnetization of the mobile charge carriers and serves as another confirmation for the presence of localized magnetic moments. Indeed, the consideration of the relevant energy scales, these are Fermi energy (*n* × 0.8 meV, where *n* is the charge carrier density in units 10^11^ cm^−2^), Zeeman energy (0.12 meV T^−1^) and the thermal energy (0.086 meV K^−1^), makes apparent that the electron system cannot reach the saturation of the magnetization in the magnetic fields of the experiment.

Since *μ*_B_ in such a description is temperature-dependent, the relation 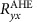
(*B*)=*γ*(*T*)*M*_*z*_=*γ*(*T*)*gμ*_B_(*T*)*JB*_*J*_(g*μ_B_(*T*)*JB*/*k*_B_*T*) is valid, where *B*_*J*_(*x*) is the Brillouin function. It is now instructive to extract the temperature dependence of the magnetic susceptibility *χ*. First, we note that *χ*=*N*^−1^(d*M*_z_/d*B*)_*B*→0_=*gμ*_B_(*T*)*J*
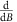
(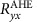
(*B*)/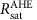)_*B*→0_, where *N* is the number of magnetic moments. Accordingly, *χ* is proportional to the slope of the experimentally obtained ratio 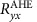
(*B*)/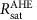 around zero field shown on the right axis in Fig. 3b and temperature-dependent *μ*_B_.

Figure 3c plots 1/*χ* versus *T* and reveals the change in the system's magnetic magnetic properties at *T*=10 K. This analysis points to a striking correlation between the AHE scalability and the magnetic properties of the heterostructure, namely, the system's magnetic property and the AHE scaling (Fig. 2f) alter at the same temperature. For *T*<10 K, the dependence 1/*χ* versus *T* can be approximated with the Curie–Weiss law with the Curie–Weiss temperature *T*_cw_ approaching 0 K.

### Comparison of studied MgZnO/ZnO heterostructures

**Figure 4:**
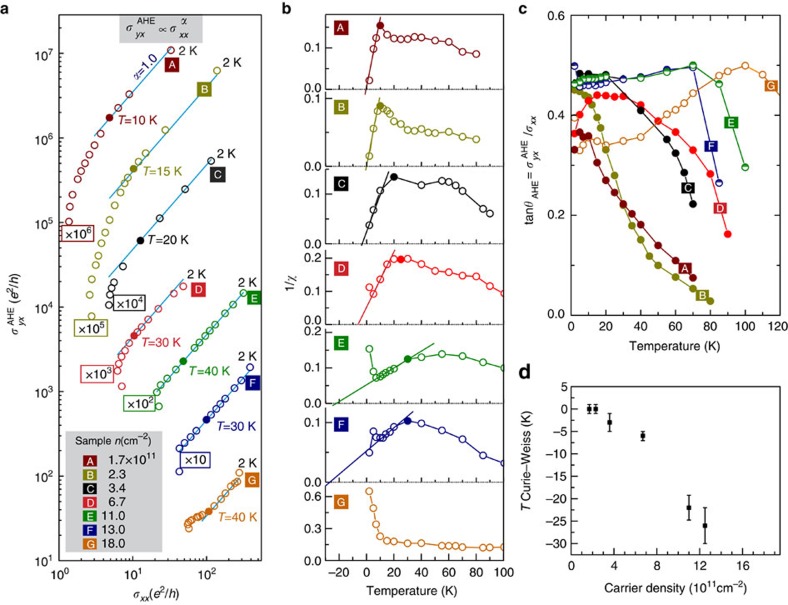
Anomalous Hall effect scaling. (**a**) Scaling of anomalous Hall effect 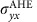
∝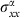 with *α*=1 (blue solid line) at low temperature is observed for structures covering a wide range of charge carrier density. For clarity of representation, 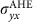 for each sample is multiplied by a factor shown in the box. *α* increases at elevated temperature. (**b**) The inverse spin-susceptibility 1/*χ* peaks at some temperature indicated by solid symbol (except the highest density sample) and suggests the change in the system's magnetic property. This transition temperature is higher for higher carrier density samples and correlates with the temperature at which *α* starts deviating from 1, except the samples E–G with the higher carrier concentration. At low temperature, 1/*χ* versus *T* dependence can be approximated with the Curie–Weiss law with a characteristic temperature *T*_cw_. (**c**) Temperature dependence of AHE angle for all samples. The AHE angle lies in the range between tan(*θ*_AHE_)=0.3 and tan(*θ*_AHE_)=0.5 at *T*=2 K, indicating a non-vanishing AHE at low temperature. (**d**) *T*_cw_ increases with the increasing carrier density. Error bar is given by the uncertainty with which the linear dependence 1/*χ* versus *T* can be approximated in **b**.

All MgZnO/ZnO heterostructures covering a range of charge carrier density between 1.7 × 10^11^ and 18 × 10^11^ cm^−2^ feature qualitatively same characteristics. Figure 4a shows the scaling of 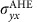 with *σ*_*xx*_, while Fig. 4b plots 1/*χ* versus *T* for all samples. The rigorous analysis of AHE conductance scaling at low temperature shows that the scaling power factor *α* lies between 0.92 and 1.08, whereas the error bar covers *α*=1.0 (Supplementary Note 7). Thus, all structures show both the AHE scaling power *α*=1 and the change in the system's magnetic property at low temperature. All data in Fig. 4a fall in one straight line when plotted on the same scale (Supplementary Note 7). It is striking that above some critical temperature, which depends on *n* and is indicated by filled symbol for samples A–D, *α* increases with the increasing temperature and simultaneously the magnetic characteristic changes, that is, the dependence 1/*χ* on *T* peaks at the critical temperature and thereafter 1/*χ* falls with *T*. Although such a correlation is particularly well pronounced in low carrier density samples, it is quite apparent that the magnetic properties are reflected in the Hall transport characteristics. Furthermore, a non-vanishing Hall angle displayed in Fig. 4c implies a persistent interaction of mobile electrons with the localized magnetic moments at all temperatures.

Finally, at low temperature, 1/*χ* dependence on *T* can be approximated with the Curie–Weiss law (except the highest carrier density sample) and we obtain *T*_cw_ at which the divergence of the uniform susceptibility is expected. Figure 4d shows the increase of the absolute value of *T*_cw_ with the carrier density, which may imply the importance of the electron correlation for the AHE appearance.

## Discussion

Our experimental results are compatible with the theoretical framework developed by GK^13^,^15^. At the moment, there is no any other theoretical model, where 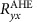
(*B*) is given by the Brillouin function to describe the polarization of localized magnetic moments by the external magnetic field. Also, GK model can explain the experimentally observed scaling between 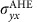 and *σ*_*xx*_, which is different from the scaling behaviour predicted by other AHE models. As mentioned above, the crucial ingredient of GK model is the coupling between the total angular momentum of localized magnetic moments **J** and the orbital motion **l** of mobile electrons, which leads to AHE as the result of the skew scattering. In such a system, the essential transport properties are characterized by (i) the total relaxation rate *τ*^−1^=*τ*^−1^(*B*, *T*) accounting for the scattering in magnetic as well as non-magnetic channels, for example, phonons and so on, and (ii) the transport correction to the cyclotron frequency *ξ*_AHE_(*B*, *T*) originating exclusively from the skew scattering. Then the Hall angle is given by (Supplementary Note 8):


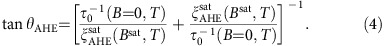


According to Fig. 4c, the AHE angle converges towards tan *θ*_AHE_≈0.4 for all samples and shows a weak temperature dependence at low temperature. In accordance with equation (4), this fact implies 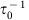
(*B*=0, *T*)≈
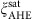
(*B*^sat^, *T*), which therefore reproduces the scaling relation 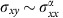 with *α*=1 identified in Figs 2f and 4a. However, when the temperature is high enough the relaxation rate 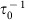, including the phonon scattering, dominates over the scattering in magnetic channel, that is, 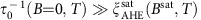, which results in a decreasing *θ*_AHE_ with increasing *T* seen in Fig. 4c.

The surprising appearance of the AHE in non-magnetic ZnO-based 2D electron system shows a new facet of many-body correlation phenomena in low-dimensional systems, which may lead to the unprecedented quantum phenomena. Moreover, the suggested Giovannini-Kondo model being compatible with the observations in ZnO can also be anticipated in other material systems^36^,^37^. Thus, ZnO lends itself as a test bed to explore the effects of localized impurities and phonon scattering on the AHE appearance in low disorder 2D electron systems.

## Methods

### Sample summary

Table 1 describes the MgZnO/ZnO heterostructures that are used in the current work.

### Data availability

The data that support the findings of this study are available from the corresponding author upon request.

## Additional information

**How to cite this article:** Maryenko, D. *et al*. Observation of anomalous Hall effect in a non-magnetic two-dimensional electron system. *Nat. Commun.* **8,** 14777 doi: 10.1038/ncomms14777 (2017).

**Publisher's note**: Springer Nature remains neutral with regard to jurisdictional claims in published maps and institutional affiliations.

## Supplementary Material

Supplementary Information Supplementary Figures, Supplementary Notes and Supplementary References

## Figures and Tables

**Figure 1:**
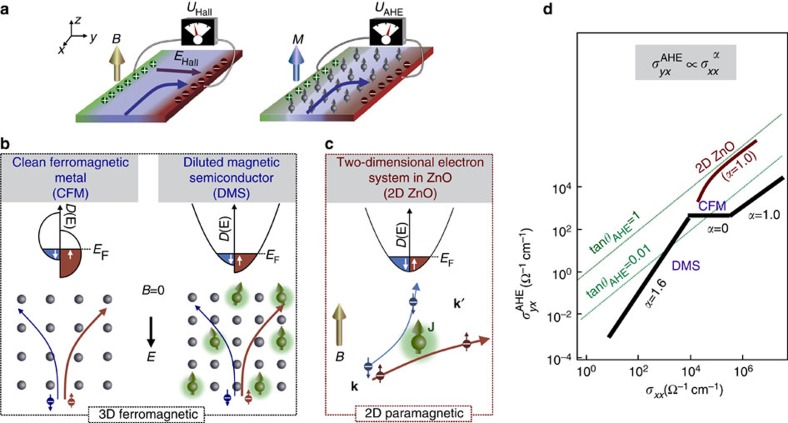
Concept of anomalous Hall effect. (**a**) Hall voltage is built in the magnetic field *B* due to the Lorentz force (left panel) and due to the spin–orbit coupling in ferromagnetic material with the total magnetization *M* (right panel). (**b**) Appearance of anomalous Hall effect: electrons are deflected in the mean field of spontaneously ordered magnetic moments. (**c**) Anomalous Hall effect in a paramagnetic system, such as ZnO, is brought about by the spin-dependent electron scattering on localized magnetic moment **J**. (**d**) Schematic representation of anomalous Hall effect scaling behaviour found in various materials^6^,^7^.

**Table 1:** Samples summary.

**Sample**	***n*** **(10**^**11**^** cm**^**−2**^**)**	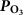 **(Torr)**	***T***_**growth**_ **(°C)**	***d***_**MgZnO**_ **(nm)**
273	3.4	2 × 10^6^	750	300
429	6.7	6 × 10^6^	750	140
430	13	6 × 10^6^	750	130
431	11	6 × 10^6^	750	140
443	18	1 × 10^5^	750	230
454	1.7	1 × 10^5^	750	250
504	2.3	2 × 10^5^	750	780

*n* is the charge carrier density at *T*=2 K, 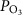 is the ozone pressure for the growth, *T*_growth_ is the growth temperature and *d*_MgZnO_ is the thickness of MgZnO layer. All heterostructures are grown on ZnO substrate.
