# Supplementary material for: Observation of anomalous Hall effect in a non-magnetic two-dimensional electron system
Source: Nat Commun. 2017 Mar 16;8:14777. doi: 10.1038/ncomms14777 (PMC5357314; doi:10.1038/ncomms14777)
Supplement: Supplementary Information — Supplementary Figures, Supplementary Notes and Supplementary References [file ncomms14777-s1.pdf]

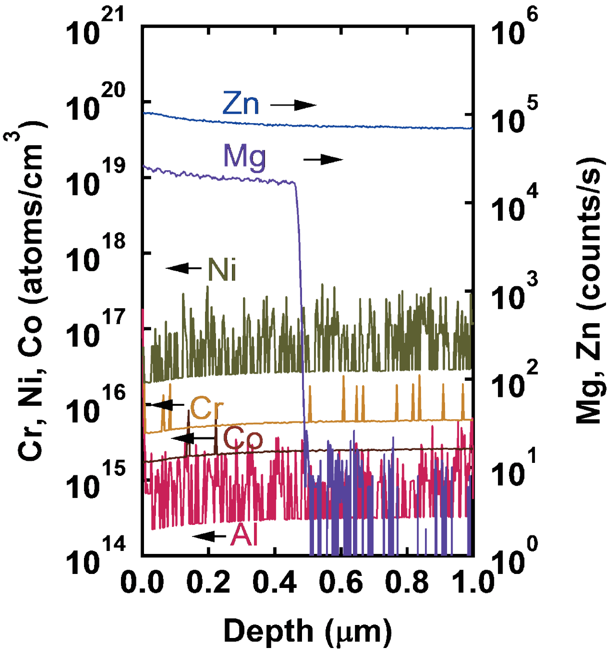


**Supplementary Figure** 1. SIMS spectra for a typical MgZnO/ZnO heterostructure

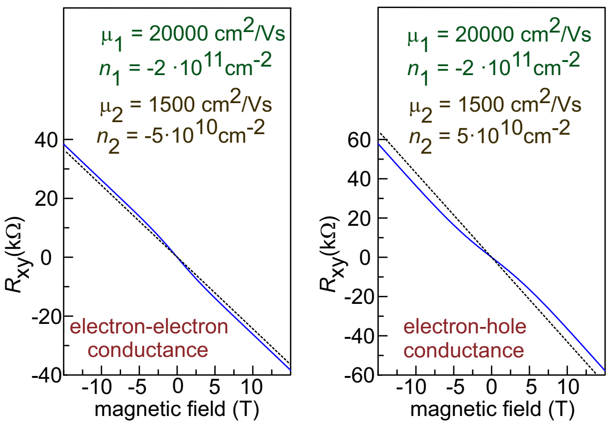


**Supplementary Figure** 2. **Two band conductance model (a)** Hall effect for two band conductance with electrons as mobile carriers in each channel. **(b)** Hall effect for two band conductance with electrons in one channel and holes in the other channel. Note, the difference in the curvature of Hall effect for both cases.

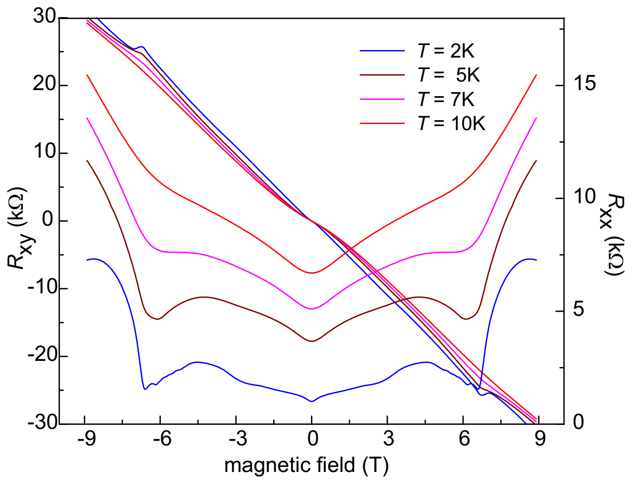


**Supplementary Figure** 3. Development of quantum Hall effect at Landau level filling factor $\nu$=1 with temperature.

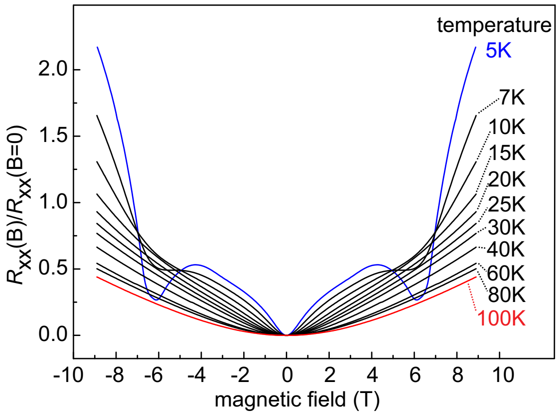


**Supplementary Figure** 4. Magnetoresistance $R_{\rm xx}$ at several temperatures.

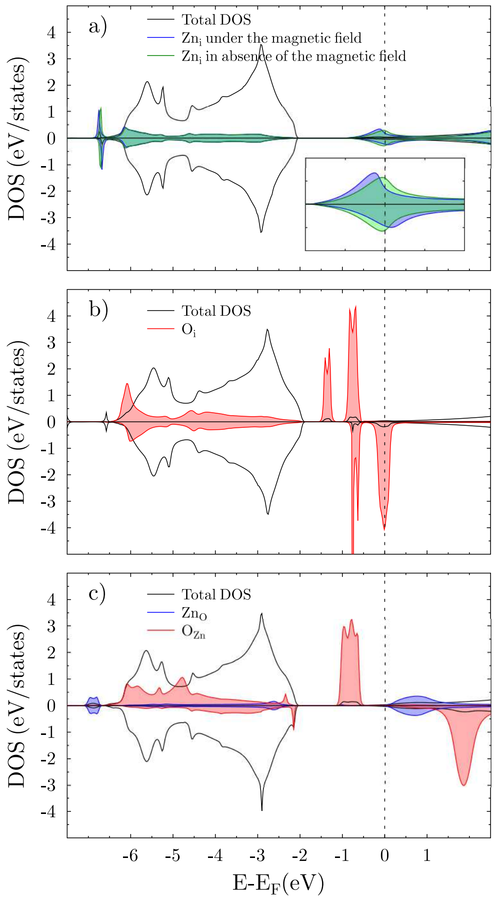


**Supplementary Figure** 5. The total spin polarized density of states and the DOS of possible point defects in ZnO matrix (1% of defect concentration): (a) Zn at tetrahedral interstitial positions under an applied magnetic field; (b) O at tetrahedral intrstitail positions; (c) antisite defects, $O_{Zn}$ and $Zn_O$. Magnetic field is zero in panels (b) and (c). The DOS of defects is shown unweighted with the concentration. Here the positive DOS is for the majority spin channel and the negative DOS is for the minority spin channel.

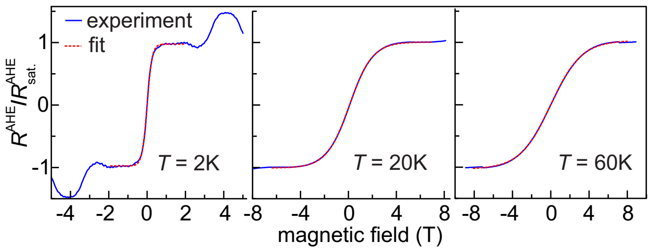


**Supplementary Figure** 6. The shape of the AHE can be well described with the Brillouin function for all temperatures.

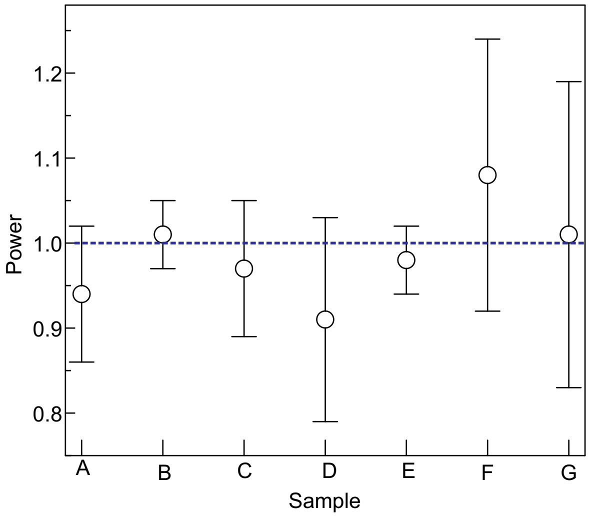


**Supplementary Figure** 7. Analysis of power factor in scaling of the anomalous Hall effect conductance.

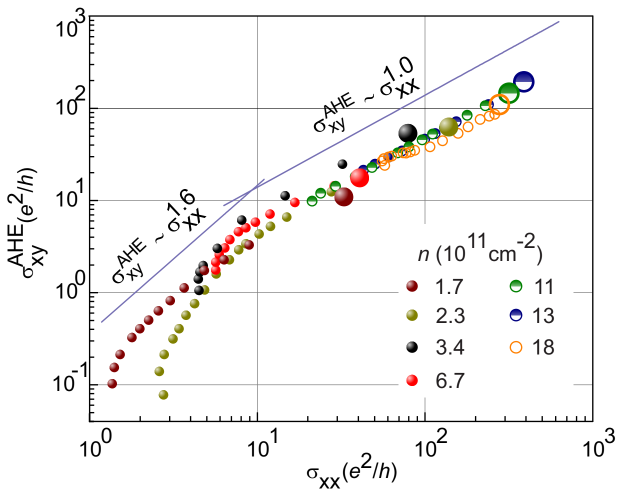


**Supplementary Figure** 8. The AHE scaling for all samples plotted in the same scale. All data fall in one straight line. Large symbols indicate the data taken at $T$=2K.

**Supplementary Note 1: Impurity characterization**

Supplementary Figure 1 shows SIMS spectra of several possible impurities for a standard high mobility MgZnO/ZnO heterostructure. Cr and other possible impurities are under the detection limit of the SIMS measurement. Therefore we can rule out the presence of other chemical elements and favor the epitaxial defects to be the source of the localized magnetic moments.

**Supplementary Note 2: Two-band conductance model**

A non-linear Hall effect is frequently a signature for a multiband conductance. We consider this scenario and show here that $R_{\mathrm{yx}}$ displayed in Fig. 2a cannot be explained with two band conductance. Each band is characterized by a charge carrier density $n$ and a mobility $\mu$. In order to account for the sign of the charge ('+' for holes and '-' for electrons) , we consider $n < 0$ for electron carriers and $n > 0$ for hole carriers. Then the Hall resistance is given by:

$$R_{\mathrm{yx}} = \frac{\mu_1^2 n_1 + \mu_2^2 n_2 + (\mu_1\mu_2 B)^2(n_1+n_2)}{e[(\mu_1|n_1| + \mu_2|n_2|)^2 + (\mu_1\mu_2 B)^2(n_1+n_2)^2]} B \tag{1}$$

We consider the parameters, which closely describe our Hall effect data, and plot $R_{\mathrm{yx}}$ calculated with the Suppl. Eq. (1) in Suppl. Fig. 2. Note, that panel (a) displays the case that electrons are charge carriers in both channels, e.g. $n_1 < 0$ and $n_2 < 0$. Panel (b) shows $R_{\mathrm{yx}}$ when only the sign of $n_2$ is changed; that is the electron conductance in one channel and hole conductance in the other channel. The dashed lines are guide to the eye to visualize the difference in $R_{\mathrm{yx}}$ bending for both cases. Thus, panel (b) reflects $R_{\mathrm{yx}}$ bending of our experimental data in Fig. 2a, implying electron-hole conductance. However, the realization of hole conductance in a wide gap ZnO semiconductor is a challenging task [1]. Moreover, the valence band discontinuity at the interface does not confine the holes. Thus, since the non-linearity as observed in our experiment can be reproduced only by assuming a parallel electron and hole conductance, we suggest that our observation implies another instance for the non-linearity; it is the anomalous Hall effect.

**Supplementary Note 3: Correction of the conductance due to electron-electron interaction**

An electron-electron interaction contributes to the field dependence of $\sigma_{\mathrm{xx}}$. Its contri-

bution in case of a diffusive transport is associated with spin effect and can be written as:

$$\delta\sigma_{\rm xx}(B,T) = \frac{e^2}{4\pi^2\hbar}\lambda_\sigma \int_0^\infty d\omega \frac{\partial^2\left(\omega\coth\frac{\omega}{2T}\right)}{\partial\omega^2} \times \left[\ln(\omega+\omega_s)+\ln(|\omega-\omega_s|)-2\ln(\omega)\right] \quad (2)$$

Here, $\hbar$=$k_B$=1 and $\omega_s = g\mu_B B$ and

$$\lambda_\sigma = 4\left[1-2\frac{(1+1/2F)\ln(1+1/2F)}{F}\right] \quad (3)$$

In the limit of strong magnetic field, i.e. $\omega_s/T > 1$, the integral becomes $\ln(\omega_s/T)$, whereas at low field, i.e. $\omega_s/T < 1$, it is approached as $0.084(\omega_s/T)^2$ [2].

Here, we employ a notation $\sigma^0_{\rm xx}$ and $\sigma^0_{\rm yx}$ indicating the sample conductance parameters without electron-electron correction. Since only $\sigma_{\rm xx}$ received a correction due to electron-electron interaction, $R_{\rm yx}$ becomes:

$$R_{\rm yx} = \frac{\sigma^{(0)}_{\rm yx}}{(\sigma^{(0)}_{\rm yx})^2+(\sigma^{(0)}_{\rm xx}+\delta\sigma_{\rm xx})^2} \approx \frac{\sigma^{(0)}_{\rm yx}}{(\sigma^{(0)}_{\rm yx})^2+(\sigma^{(0)}_{\rm xx})^2+2\sigma^{(0)}_{\rm xx}\delta\sigma_{\rm xx}} \quad (4)$$

The latter equation is due to the fact that $\delta\sigma_{\rm xx} \ll \sigma^{(0)}_{\rm xx}$. This equation is then modified to:

$$R_{\rm yx} = \frac{\sigma^{(0)}_{\rm yx}}{(\sigma^{(0)}_{\rm yx})^2+(\sigma^{(0)}_{\rm xx})^2}\frac{1}{1+2\frac{\sigma^{(0)}_{\rm xx}}{(\sigma^{(0)}_{\rm yx})^2+(\sigma^{(0)}_{\rm xx})^2}\delta\sigma_{\rm xx}} = R^{(0)}_{\rm yx}\frac{1}{1+2R^{(0)}_{\rm xx}\delta\sigma^{(0)}_{\rm xx}} \approx R^{(0)}_{\rm yx}(1-2R^{(0)}_{\rm xx}\delta\sigma_{\rm xx}) \quad (5)$$

Thus the correction to the Hall resistance due to electron-electron interaction becomes:

$$\Delta R^{\rm I}_{\rm yx} = R_{\rm yx} - R^{(0)}_{\rm yx} = -2R^{(0)}_{\rm yx}R^{(0)}_{\rm xx}\delta\sigma_{\rm xx} \quad (6)$$

In order to visualize the field dependence of this correction, we approximate the Hall effect as $R_{\rm yx} = \alpha+\beta B$ and $R_{\rm xx} = \gamma+\theta B^2$. Then the correction has the following field dependence in the limit of high field:

$$\Delta R^{\rm I}_{\rm yx} = (\alpha+\beta B)(\gamma+\theta B^2)\ln B \quad (7)$$

Obviously, this correction is more pronounced as the magnetic field is larger. Hence the correction increases in the magnetic, which is the opposite to the saturation behavior shown

in the main text in Fig.2b. Thus the electron-electron interaction cannot explain the non-linearity as observed in our experiment.

## Supplementary Note 4: Additional magnetotransport data

### Magnetotransport between T=2K and T=10K

Supplementary Figure 3 depicts the development of Landau level filling factor $\nu = 1$ by displaying the temperature dependence of $R_{\mathrm{xx}}$ and $R_{\mathrm{yx}}$ for sample presented in Figs.2 and 3 of the main text.

### Positive magnetoresistance

ZnO heterostructures show a positive magnetoresistance. Supplementary Figure 4 exemplifies $R_{\mathrm{xx}}$ field dependence at several temperatures for the samples discussed in Figs. 2 and 3 of the main text.

## Supplementary Note 5: Possible origin for magnetism in MgZnO/ZnO heterostructures

To elucidate the origin of spin polarized carriers at the MgZnO/ZnO interface, we performed extensive first-principles calculations within the density functional theory (DFT). The density functional theory provides an adequate description of many properties of ZnO and MgZnO except of excited state properties such as the size of the band gap. The latter is the lack of the DFT, which is designed by construction only for ground state properties. However, structural relaxations, formation energy, magnetic moments, densities of states for core and valence electrons are usually reproduced correctly. For our calculations we used a self-consistent Green function method within the multiple scattering theory, which is specially designed for semi-infinite systems such as surface and interfaces [3, 4]. Disorder effects were simulated using a coherent potential approximation as it formulated within the multiple scattering formalism [5, 6]. This approach has proven to be reliable to describe structural, electronic and magnetic properstis of ZnO and related materials [7–17].

Among the carriers, which can participate in spin dependent transport at the ZnO/ZnMgO

we identified the following point defects acting as donnors, which can arise very likely during the growth process: (i) Zn at interstitial octahedral sites ($Zn_i$); (ii) O at interstitial octahedral sites ($O_i$) calculations ($O_i$); (iii) antisite defects ($O_{Zn}$, $Zn_O$) [18]. The latter have a quite high formation energy, but (i) and (ii) are also possible in a non-equilibrium growth process. $Zn_i$ is not magnetic but can be magnetized in an external magnetic field (Fig.5(a)). $O_i$ and $O_{Zn}$ posses magnetic moments of 1.07 $\mu_B$ and 1.99 $\mu_B$, respectively (Suppl. Fig. 5(b,c)). The magnetic moments are formed by localized unpaired electrons. Since the DFT fails to describe correctly excited state properties, the band gap was underestimated in our calculations (1.6 eV vs. 3.25 eV).

**Supplementary Note 6: Brillouin function**

To describe the shape of the AHE we use the Brillouin function:

$$B_J(x) = \frac{2J+1}{2J}\coth(\frac{2J+1}{2J}x) - \frac{1}{2J}\coth(\frac{1}{2J}x) \tag{8}$$

where

$$x = \frac{g\mu_B JB}{k_B T} \tag{9}$$

In our model we assume a simple scenario. The magnetic moments are characterized by $J = 1/2$ and $g$-factor 2. Thus the moment $\mu_B$ remains the only fitting parameter in Suppl. Eq. 9. The fitting is performed for all temperatures of the experiment (between 2K and 70K) and $\mu_B$ is deduced. The examples of fitting at three representative temperatures are shown in Suppl. Fig. 6. The temperature dependence of $\mu_B$ is plotted in Fig. 3b in the main text. Both large $\mu_B$ values and temperature dependent $\mu_B$ are obtained for all studied samples. The assumption of a different parameter set with $J > 1/2$, e.g. $J = 3/2$, cannot reproduce the AHE shape for all temperature of the experiment. We therefore conclude that the as-chosen parameters describe the best the system's behavior and implies the superparamagnetic behavior. Furthermore, the fact, that the Brillouin function describes so well the AHE shape at all temperatures and for all samples, strongly suggests that the AHE is related to the localized magnetic moments with $J = 1/2$ and $g = 2$. Such a parameter set is consistent with the assumption that the local magnetic moments are electrons localized at the defects of the epitaxial film.

## Supplementary Note 7: anomalous Hall effect scaling

### Scaling analysis

We analyzed the scaling of the anomalous Hall effect conductance at low temperature for all samples summarized in Fig. 4 of the main text. We used the fitting function:

$$\sigma_{\mathrm{yx}}^{\mathrm{AHE}} = a \cdot \sigma_{\mathrm{xx}}^{\alpha} \tag{10}$$

to deduce the scaling power factor $\alpha$. The result is shown in Suppl. Fig. 7; $\alpha$=1.0 value is covered by all samples within the error bar.

### Scaling summary of all samples

When all data in Fig. 4a in the main text are plotted on the same scale, they all fall in one straight line as shown in Suppl. Fig. 8.

## Supplementary Note 8: Giovannini-Kondo model for anomalous Hall effect

### Hall angle and scaling

In the Giovannini-Kondo model [19–21], the skew scattering originates from the interaction between localized magnetic moment **J** and orbital momentum **l** of the conduction electron. The interaction between a conduction electron and a localized magnetic moment is defined by the Hamiltonian

$$H_{int} = V_0 + J_{ex}\mathbf{J} \bullet \sigma + \lambda \mathbf{J} \bullet \mathbf{l} \; . \tag{11}$$

Here, $V_0$ is a term describing the spin independent scattering on impurities and $J_{\mathrm{ex}}$ is a conventional exchange term describing interaction between localized magnetic moment **J** and spin of conduction electron $\sigma$. Both components do not lead to skew scattering. The skew scattering arises from the third term which leads to the orbital exchange interaction

$$H_{int}^{skew} \sim \lambda i (\mathbf{k} \times \mathbf{k}') \bullet \mathbf{J} \; . \tag{12}$$

According to theory of AHE [21] arising due to scattering on magnetic impurities in paramagnetic system the transport properties of the system can be expressed in terms of two characteristic rates having dimension of the inverse time. The first one is the total relaxation rate $\tau^{-1} = \tau^{-1}(B,T)$ accounting for scattering in magnetic as well as non-magnetic channels, e.g. phonons, etc. The second one, $\xi_{\rm AHE}(B,T)$, is the transport correction to the cyclotron frequency

$$\omega_\sigma(H) = eB/mc + \xi_{\rm AHE}(B,T) \qquad (13)$$

originating exclusively from skew scattering term (12). Field dependence of the skew scattering rate is proportional to the Brillouin function. Here $m$, $e$, and $c$ is the mass, electron charge, and light velocity, respectively.

These two rates are considerably entangled in the expressions for the conductivity components

$$\begin{pmatrix} \sigma_{xx} \\ \sigma_{yx} \end{pmatrix} = \theta \begin{pmatrix} \tau^{-1} \\ \omega_\sigma \end{pmatrix} , \qquad (14)$$

where $\theta = \alpha/[(\tau^{-1})^2 + \omega_\sigma^2]$, and $\alpha = ne^2/mc$ depends on the carriers concentration $n$. However, these rates can be highlighted by the experimental procedure described below.

The resistivity components $(R_{xx}, R_{\rm yx})$ can be expressed in terms of conductivity components $(\sigma_{\rm xx}, \sigma_{\rm yx})$

$$\begin{pmatrix} R_{xx} \\ R_{\rm yx} \end{pmatrix} = \begin{pmatrix} \sigma_{xx} \\ \sigma_{\rm yx} \end{pmatrix} \frac{1}{\sigma_{xx}^2 + \sigma_{\rm yx}^2} . \qquad (15)$$

Similar expression, with $\sigma_{xx} \leftrightarrow R_{xx}$ and $\sigma_{xy} \leftrightarrow R_{xy}$, is valid to express $(\sigma_{xx}, \sigma_{xy})$ in terms of $(R_{xx}, R_{xy})$.

The longitudinal resistivity, measured at zero field, singles out the total relaxation rate

$$R_{xx}(B=0) = \frac{1}{\sigma_{xx}(B=0)} = \frac{\tau_0^{-1}(T)}{\alpha} , \qquad (16)$$

where $\tau_0^{-1}(T) = \tau^{-1}(B=0,T)$. Due to the specific structure of Eqs. (14) and (15) the transverse component of resistivity gains a simple form

$$R_{\rm yx} = \frac{\theta\omega_\sigma}{\theta^2(\tau^{-1})^2 + \theta^2\omega_\sigma^2} = \frac{\omega_\sigma(B,T)}{\alpha} . \qquad (17)$$

To highlight the transport correction to the cyclotron frequency one has to extract ordinary Hall resistivity to get the AHE contribution

$$R_{yx}^{AHE} = \frac{1}{\alpha}\left[\omega_\sigma - \left(\frac{d\omega_\sigma}{dB}\right)_{B\to\infty} B\right] \qquad (18)$$

and, then, take the experimental value of AHE resistivity $R_{yx}^{AHE,sat}$ at saturation

$$R_{yx}^{AHE,sat} = \frac{\xi_{\rm AHE}(B \to \infty, T)}{\alpha} = \frac{\xi_{\rm AHE}^{\rm sat}(T)}{\alpha} \; . \tag{19}$$

Then, if the conductivity $\sigma_{yx}^{AHE,sat}$ is determined by inverting Eq. (15) through the resistivity components $(R_{xx}(B=0), R_{yx}^{AHE,sat})$, one gets

$$\sigma_{\rm yx}^{AHE,sat} = \frac{\alpha \xi_{\rm AHE}^{\rm sat}(T)}{(\tau_0^{-1}(T))^2 + \xi_{\rm AHE}^{\rm sat}(T)^2} \; . \tag{20}$$

Using Eq. (16) and introducing Hall angle

$$\tan\theta_{AHE} = \frac{\sigma_{yx}^{AHE,sat}}{\sigma_{xx}(B=0)} \tag{21}$$

one gets

$$\tan\theta_{AHE} = \left[\frac{\tau_0^{-1}(T)}{\xi_{\rm AHE}^{\rm sat}(T)} + \frac{\xi_{\rm AHE}^{\rm sat}(T)}{\tau_0^{-1}(T)}\right]^{-1} \; . \tag{22}$$

Hall angle decreases when temperature increases because the total relaxation rate, including phonon scattering, dominates at high temperatures. Therefore, one can assume that at high temperatures

$$\tau_0^{-1}(T^{HIGH}) \gg \xi_{\rm AHE}^{\rm sat}(T^{HIGH}) \; . \tag{23}$$

Equation (22) implies that

$$\frac{\tau_0^{-1}(T)}{\xi_{\rm AHE}^{\rm sat}(T)} = \frac{1}{2\tan\theta_{AHE}} \pm \sqrt{\left[\frac{1}{2\tan\theta_{AHE}}\right]^2 - 1} \; . \tag{24}$$

Therefore, low temperature values of Hall angle $\tan\theta_{AHE} \approx 0.4 \sim 0.5$ necessarily mean that at low temperatures

$$\tau_0^{-1}(T^{LOW}) \approx \xi_{\rm AHE}^{\rm sat}(T^{LOW}) \; . \tag{25}$$

We note that $\tan\theta_{AHE}$ is temperature independent in some range of low temperatures. This implies low temperature scaling relation $\sigma_{yx} \sim \sigma_{xx}^{\alpha}$ with $\alpha = 1$. In the high temperature regime, when total relaxation rate $\tau_0^{-1}(T^{HIGH})$ growth faster than the transport correction to the cyclotron frequency $\xi_{\rm AHE}^{\rm sat}(T^{HIGH})$, the scaling behavior does not hold.

**Consequences of the model**

Magnetic susceptibility, extracted from transport experimental data, demonstrate significant change of temperature dependence at $T_c = 10$K. In the case of ferromagnetic or

metamagnetic phase transition there is a known resistivity anomaly peaking near the magnetic transition temperature. There is no true transition in our sample where positions of magnetic moments are disordered though the peak in the scattering rate on magnetic subsystem is possible if at different temperatures system is close to different magnetic phases. This component, $\delta\rho_{peak}(T-T_c)$, contributes both to magnetic and nonmagnetic scattering rates, although with different coefficients: $\xi^{\rm sat}_{\rm AHE}(T) = A_1\delta\rho_{peak}(T-T_c)$ and $\tau_0^{-1}(T) = A_2\delta\rho_{peak}(T-T_c) + CT$ . Here, the contribution $CT$ to the total relaxation rate is the high temperature Bloch-Grüneisen phonon contribution. Finally, the scaling expression is

$$\sigma_{yx}^{EXP} \approx \sigma_{xx}(H=0)\frac{A_1\delta\rho_{peak}(T-T_c)}{A_2\delta\rho_{peak}(T-T_c)+CT} \,. \tag{26}$$

First term of denominator is larger than the second term at low temperatures leading to $\sigma_{yx}^{EXP} \sim \sigma_{xx}(H=0)$ scaling. On the other hand the scaling power gradually increases when the term $CT$ becomes larger than the contributing $A_2\delta\rho_{peak}(T-T_c)$, which decreases with temperature at $T > 10$K.

**Summary of main points of Giovannini-Kondo model**

- The electron system does not need to be spin polarized. This is the main difference to other AHE models. Our experimental findings show that the electron system is not spin-polarized, which is confirmed by the observation of multiple spin-resolved Lanau level coincidence events (Nature Physics 11, 347 (2015) and Phys. Rev. B 90, 245303 (2014)).

- The field dependence of the AHE resistance on the magnetic field can be described with the Brillouin function

- A positive magnetoresistance can be expected. Note, that diluted magnetic semiconductors show negative magnetoresistance

- The angle of anomalous Hall effect is directly derived.

- Giovannini-Kondo model can account for some temperature dependent properties, e.g. scaling or AHE angle, in a reasonable agreement with experiment.

## SUPPLEMENTARY REFERENCES


[1] Tsukazaki, A. *et al.* Repeated temperature modulation epitaxy for $p$-type doping and light-emitting diode based on ZnO. *Nat. Materials* **4**, 42–46 (2005).

[2] Lee, P. A. & Ramakrishnan, T. V. Disordered electronic systems. *Rev. Mod. Phys.* **57**, 287–337 (1985).

[3] Lüders, M., Ernst, A., Temmerman, W. M., Szotek, Z. & Durham, P. J. Ab initio angle-resolved photoemission in multiple-scattering formulation. *Journal of Physics: Condensed Matter* **13**, 8587 (2001).

[4] Geilhufe, M. *et al.* Numerical solution of the relativistic single-site scattering problem for the Coulomb and the Mathieu potential. *Journal of Physics:Condensed Matter* **27**, 435202 (2015).

[5] Soven, P. Coherent-potential model of substitutional disordered alloys,. *Phys. Rev.* **156**, 809–813 (1967).

[6] Gyorffy, B. L. Coherent-potential approximation for a nonoverlapping-muffin-tin-potential model of random substitutional alloys. *Phys. Rev. B* **5**, 2382–2384 (1972).

[7] Maznichenko, I. V. *et al.* Structural phase transitions and fundamental band gaps of $Mg_xZn_{1-x}O$ alloys from first principles. *Phys. Rev. B* **80**, 144101 (2009).

[8] Meyerheim, H. L. *et al.* Wurtzite-type CoO nanocrystals in ultrathin ZnCoO films. *Phys. Rev. Lett.* **102**, 156102 (2009).

[9] Khalid, M. *et al.* Defect-induced magnetic order in pure ZnO films. *Phys. Rev. B* **80**, 035331 (2009).

[10] Adeagbo, W. A., Fischer, G., Ernst, A. & Hergert, W. Magnetic effects of defect pair formation in ZnO. *Journal of Physics: Condensed Matter* **22**, 436002 (2010).

[11] Bekenov, L. V. *et al.* Electronic and magnetic properties of $(Zn_{1-x}V_x)O$ diluted magnetic semiconductors elucidated from X-ray magnetic circular dichroism at V $L_{2,3}$ edges and first-principles calculations. *Phys. Rev. B* **84**, 134421 (2011).

[12] Fischer, G. *et al.* Room-temperature $p$-induced surface ferromagnetism: First-principles study. *Phys. Rev. B* **84**, 205306 (2011).

[13] Esquinazi, P., Hergert, W., Spemann, D., Setzer, A. & Ernst, A. Defect-induced magnetism in solids. *IEEE Transactions on Magnetics* **49**, 4668–4668 (2013).

[14] Adeagbo, W. A., Thomas, S., Nayak, S. K., Ernst, A. & Hergert, W. First-principles study of uniaxial strained and bent ZnO wires. *Phys. Rev. B* **89**, 195135 (2014).

[15] Meyerheim, H. L. *et al.* Wurtzite structure in ultrathin ZnO films on Fe(110): Surface X-ray diffraction and ab initio calculations. *Phys. Rev. B* **90**, 085423 (2014).

[16] Lorite, I. *et al.* Advances in methods to obtain and characterise room temperature magnetic ZnO. *Applied Physics Letters* **106**, 082406 (2015).

[17] Fischer, G. *et al.* Ab initio study of the $p$ -hole magnetism at polar surfaces of ZnO: the role of correlations. *Journal of Physics: Condensed Matter* **28**, 016003 (2016).

[18] Janotti, A. & de Walle, C. G. V. Fundamentals of zinc oxide as a semiconductor. *Rep. Prog. Phys.* **72**, 126501 (2009).

[19] Kondo, J. Anomalous Hall effect and magnetoresistance of ferromagnetic metals. *Progress of Theoretical Physics* **27**, 772–792 (1962).

[20] Maranzana, F. E. Contributions to the theory of the anomalous Hall effect in ferro- and antiferromagnetic materials. *Phys. Rev.* **160**, 421–429 (1967).

[21] Giovannini, B. Skew scattering in dilute alloys. I. The Kondo model. *Journal of Low Temperature Physics* **11**, 489–507 (1973).
